# A Bayesian analysis of longitudinal farm surveys in Central Malawi reveals yield determinants and site-specific management strategies

**DOI:** 10.1371/journal.pone.0219296

**Published:** 2019-08-08

**Authors:** Han Wang, Sieglinde S. Snapp, Monica Fisher, Frederi Viens

**Affiliations:** 1 Department of Statistics and Probability, Michigan State University, East Lansing, MI, United States of America; 2 Department of Plant, Soil and Microbial Sciences, Michigan State University, East Lansing, MI, United States of America; 3 International Centre of Insect Physiology and Ecology, Nairobi, Kenya; Assam University, INDIA

## Abstract

Understanding the challenges to increasing maize productivity in sub-Saharan Africa, especially agronomic factors that reduce on-farm crop yield, has important implications for policies to reduce national and global food insecurity. Previous research on the maize yield gap has tended to emphasize the size of the gap (theoretical vs. achievable yields), rather than what determines maize yield in specific contexts. As a result, there is insufficient evidence on the key agronomic and environmental factors that influence maize yield in a smallholder farm environment. In this study, we implemented a Bayesian analysis with plot-level longitudinal household survey data covering 1,197 plots and 320 farms in Central Malawi. Households were interviewed and monitored three times per year, in 2015 and 2016, to document farmer management practices and seasonal rainfall, and direct measurements were taken of plant and soil characteristics to quantify impact on plot-level maize yield stability. The results revealed a high positive association between a leaf chlorophyll indicator and maize yield, with significance levels exceeding 95% Bayesian credibility at all sites and a regression coefficient posterior mean from 28% to 42% on a relative scale. A parasitic weed, *Striga asiatica*, was the variable most consistently negatively associated with maize yield, exceeding 95% credibility in most cases, of high intensity, with regression means ranging from 23% to 38% on a relative scale. The influence of rainfall, either directly or indirectly, varied by site and season. We conclude that the factors preventing *Striga* infestation and enhancing nitrogen fertility will lead to higher maize yield in Malawi. To improve plant nitrogen status, fertilizer was effective at higher productivity sites, whereas soil carbon and organic inputs were important at marginal sites. Uniquely, a Bayesian approach allowed differentiation of response by site for a relatively modest sample size study (given the complexity of farm environments and management practices). Considering the biophysical constraints, our findings highlight management strategies for crop yields, and point towards area-specific recommendations for nitrogen management and crop yield.

## Introduction

Yield gaps in African smallholder agriculture are pervasive and large [1]. The yields achieved on the vast majority of African farms is 10–30% of genetic yield potential [2]. Yield-limiting factors have been identified, such as environment, sub-optimal planting in terms of timing and spacing, as well as deficiencies in soil nutrients, moisture, and damage from weeds and pests [3–6]. Agricultural economists also investigate yield gaps, and commonly emphasize market prices, farmer education, and related socio-economic factors thought to influence on-farm production [7]. There are many challenges to carrying out effective diagnostic analysis of yield gaps, and often the focus has been on the size of the gap—theoretical vs achievable yields [8, 9]. Yet if research priorities and agronomic recommendations are to address farm-level constraints, there is urgent need for evidence-based assessment of the main determinants of yield, in specific contexts [10]. Further, to ensure relevance of technical advice in a complex and changing world, statistical approaches are required that consider uncertainity as part of understanding yield determinants [4, 11].

The present study assesses the main determinants of maize yield by applying a Bayesian approach to a unique survey dataset from Central Malawi. Crop simulation models are often used for yield gap analysis and are well suited to providing insights into yield potential and technology response to weather variability; however, this approach does not reveal the drivers of yield gaps [12, 13]. Field experimentation, as generally implemented, also has flaws. In particular, trials are often run under conditions that are not representative of on-farm conditions. Smallholders in sub-Saharan Africa often have marginal soils, practice variable and less intensive management, and face weed, disease, insect, and other pest problems [1, 14]. The disconnect between soil conditions on research stations and those on smallholder farms is illustrated by a country-wide assessment in Malawi, where soil organic matter levels on research stations were 1.5 to 2 times as high as those observed on smallholder farms [15]. Researchers generally choose a field site and invest resources, so as to ensure a homogenous and uniform environment within which to evaluate a practice or address a specific research question. Thus field research sites tend to be flat, uniform and high-potential, given that conventional research experimentation generally tests one or two component practices while controlling other sources of variability [16]. In contrast, there is growing interest in action types of research and extension that involve mixed-methods approaches such as surveys linked to on-farm experimentation, to support informed understanding of complex interactions, tradeoffs and local adaptation [17, 18].

This study is the first to examine the determinants of maize yield using a Bayesian approach. One advantage of this approach over classical frequentist statistics is improved accuracy and credibility of the estimated parameters, because the Bayesian analysis incorporates background knowledge from domain specialists [19]. Second, Bayesian statistics have interpretive advantages as Bayesian credible intervals are straightforward to interpret by non-statisticians. A third advantage is that a Bayesian analysis always provides precise answers in the form of posterior probabilities, no matter what the model nor the data is. The model’s various uncertainties are all quantifiable and readily reportable. In particular, no reliance on large samples is needed, an important aspect given that farm survey sample sizes are often limited relative to the complexity of biophysical environment and farmer management decision making. Taken together, these points indicate that Bayesian statistics could be a powerful data analysis tool for agricultural research questions, such as understanding yield determinants [11].

The overall objective of this study was to conduct a Bayesian analysis of household survey data that comprised multiple visits to focal maize plots in Central Malawi, to determine which variables influenced maize yields. Specifically, we assessed the predictive ability of time series environmental variables and management practices regarding field observations of maize yield, for specific sites. Further, we evaluated leaf chlorophyll status and parasitic weed incidence and determined area-specific predictive models for these important variables.

## Materials and methods

### Study sites

Central Malawi agriculture is dominated by mixed maize production systems with limited livestock presence, and is broadly typical of resource poor smallholder farms in Southern Africa [14]. Administrative units used by Malawi Government Extension are comprised of region, agricultural development divisions and extension planning areas (EPA). The study sites were chosen using a stratified random sampling approach, where all EPAs within the Central Malawi region were classified using the strata of marginal, moderate or mesic (based on rainfall and evapotranspiration) for plant growth. Study sites were choosen randomly to represent strata [14]. The marginal site comprises two adjacent EPAs, Golomoti and Mtakataka (referred to herein as Golomoti), two moderate potential sites were chosen, Kandeu and Nsipe, and the high potential site comprises Linthipe, with a total of 22 village clusters included within these five EPAs [14].

The marginal site of Golomoti is located near the lakeshore at a low elevation with a high evapotranspiration, with a mix of soil types, dominated by Eutric Cambisols and Eutric Fluvisols [20]. Linthipe is a high potential site, with well-distributed rainfall and a long history of maize-dominated agriculture as part of Malawi’s maize basket [6, 21]. Soils in Linthipe are primarily Ferric Luvisols [20]. Kandeu and Nsipe EPAs are medium potential sites, with soils dominated by mixed Chromic Luvisols and Orthic Ferrasols [20]. Market access also varies across study locations, with Kandeu and Nsipe being moderately remote and Golomoti and Linthipe being proximate to markets.

### Survey method

#### Survey location in space and time

Data for the study were from a panel of 320 farm households, with two maize plots per household surveyed in 2014/15 and in 2015/16, at pre-season, mid-season and at harvest. Data from a total of 1,197 plots were used in the analysis, due to a small amount of missing data. The sampled farm households have participated in a USAID-funded Africa RISING research project and include intervention, local control, and distant control households from 22 village clusters in the five EPAs. The data used in the present study are from detailed plot-level surveys of the sampled farmers, who were asked to choose two maize plots at random, which were geo-located with GPS coordinates in October of 2014 and soil sampled as described below. The same plots and farmers were then revisited and surveyed at midseason (March 2015 and 2016) and at harvest (May 2015 and 2016).

The data collected in 2014/15 and 2015/16 were longitudinal which combined socio-ecnomic information, crop production, farm management, plant and soil characteristics. The household-level data were collected with a survey instrument approved through the Michigan State University (MSU) Human Research Protection Program (HRPP) in the Office of Regulatory Affairs, following a human subjects’ protocol with informed consent obtained from all farmers, translated into local languages, and information provided on the survey based on valuntarity. Every effort was carried out to maintain confidentiality. Enumerators were trained over a one-week period, and supervised in the field by graduate students, and the data collection process included close attention to data entry and data quality control.

#### Survey data collection

Survey topics addressed include socio-economic characteristics (household size and composition, household head’s educational level) and farm-level management and focal plot practices that included labor, seed information, planting dates, and plot management and history (farmer-reported crop residue management, crops grown, timing of sowing and weeding, and fertilizer application). Soil samples were collected from maize plots using 5-cm diameter auger to sample from the 0–20 cm depth, where 8 samples were composited per plot after collection using a Z-scheme [22]. The soil sampling was thus all done on private land, following the survey consent procedure described above, supervised by the MSU HRPP. Soils were air dried, shipped to MSU laboratory and analyzed for pH, total C, and permanganate oxidizable carbon (POXC). Soil pH was measured in a 1:2 soil:water solution and total organic C determined by dry combustion in a CHNS analyzer (Costech ECS 4010, Costech Analytical Technologies, Valencia, CA). POXC was analyzed as follows: 2.5 g of air-dried soil centrifuged with 18 mL of deionized water, 2 mL of 0.2 *M* KMnO4 stock solution added and tubes shaken for exactly 2 min at 240 oscillations per minute on an oscillating shaker. Sample absorbance on an aliquot was read at 550 nm with a SpectraMax M5 microplate reader using SoftMax Pro software (Version 5.4.1, Molecular devices, Sunnyvale, CA) at exactly 10 min; we find precise timing to be critical for the POXC analytical procedure.

Midseason measurements were made assessing maize planting arrangements (including row spacing), maize leaf chlorophyll, based on Soil Plant Analysis Development (SPAD) absorbance using an atLeaf CHL instrument (Green, LLC Wilmington, DE http://www.atleaf.com). Enumerators recorded three reading replicates per plant for four plants at each of eight locations. In addition, two types of measurements were made to record the incidence of *Striga asiatica* (L.) Kuntze, commonly known as witchweed, a genus of parasitic plants. One method involved directly asking farmers if they had a striga problem on a given maize plot. Striga information was also obtained by enumerators who made 8 observations per plot at random sites along rows following a prescribed procedure; thus, for each plot, striga observations were recorded from 0 to 8. At harvest, a survey was conducted to measure maize crop yield by weighing biomass of stover and grain from three square meter plots per field, where grain was removed from cobs and grain moisture determined in the field with a Dickey John moisture tester to allow maize yield to be reported on a dry weight basis.

In summary, this study included plot-level longitudinal data on socio-economic information, crop production, farm management, plant and soil characteristics (from soil samples, leaf SPAD measurements and weighted biomass and grain after harvest).

### Statistical model

#### Modeling framework and variables

A Bayesian framework was used to estimate the statistical relation between maize yield data and data on the farmer and plot characteristics where the relationships were specified in a linear regression model. An agronomic perspective, based on expert knowledge was used to form the basis of this model, as follows:

$$Y_{ijk}=\alpha_{i}+X_{ijk}\beta_{i}+\sigma \epsilon_{ijk}.$$

where *Y*_*ijk*_ is the maize yield (in kilograms per hectare) of plot *k* managed by farm household *j* at EPA site *i*; *α*_*i*_ is the *y*-intercept of the linear regression model, which can be a constant throughout the model or can vary from EPA site to EPA site; *X* is a design matrix that incorporates all the data from the explanatory variables, i.e. the factors that may influence yield; *β*_*i*_ is a vector of regression coefficients which measure how much of the variation of maize yield is accounted for by the explanatory variables, and may also depend on the EPA site *i*; *ϵ*_*ijk*_ are independent Gaussian noise terms (having mean zero and standard deviation of one) that represent the statistical error of the model; and the standard deviation *σ*, which represents the scale of the error, and can be compared with the magnitude of the regression coefficients. When *α* and *β* depend on the EPA site index *i*, one can gauge the “random” effect of location. The two year data were pooled in the Bayesian model. Pooling the two years avoided model misspecification, and has the added benefit of increasing the study’s statistical power. Further models tested the response variable SPAD, and the parasitic weed striga, to uncover underlying key drivers of maize yield, where we expected striga to be a negative driver and SPAD a positive factor. The noise terms were assumed to be independent across models in order to minimize the number of parameters that needed to be estimated for the sake of statistical power. This avoids at least three correlation parameters. It also avoids the use of a large number of correlation parameters (hyperparameters) at the prior level in the Bayesian context, which would be needed to be consistent with investigating a system of 3 equations with 12 explanatory variables in common between any pair of models (e.g. Maize and *Striga* models), one should, at least, assume a prior correlation for pairs of regression coefficients for each explanatory variable, thus another 36 prior correlation parameters. Consequently unobserved factors that might simultaneously affect more than one model are not taken into account.

Environmental factors (rainfall and soil properties) and farmer management on maize yield which are part of the vector *X* were either continuous or binary variables, standardized (mean 0 and standard deviation 1), where continuous variables allowed for comparison of the estimated coefficients on the same scale. Rainfall data came from the Climate Hazards InfraRed Precipitation with Station (CHIRPS) resource, which is a public quasi-global rainfall dataset source starting in 1981. This is the only comprehensive precipitation data source that is available for Malawi, and has been previously validated by comparisons to local rainfall records for three of the five EPAs [14]. We included three rainfall variables in the model that record the amount of rainfall (millimeter) for the months of (a) December, January, and February (the sowing period), (b) March (the end of the rainy season), and (c) April and May (the harvest period). By measuring rainfall at three key stages of the maize growing season we account, to some degree, for the association between seasonal rainfall variability and maize yield. This use of more than one subset of semi-annual rainfall is common in certain agricultural studies and practices, including in the definition and calculation of rain-index-based crop insurance [23–25]. Regarding soil properties, POXC, which is a sensitive indicator of active soil organic C, and pH, were selected as the main soil factors in our model [26].

We also included five key farm management variables, namely ridge (row) spacing, total ridge weed biomass, fertilizer use, intercropping, and manure/compost use. Although fertilizer and SPAD were highly correlated, they both had explanatory power for maize yield, indicating that they should be included simultaneously in the model. We note that endogeneity bias is possible, particularly for the case of farmer management variables, given these are choice variables. However, we are unable to address endogeneity, because our dataset does not include suitable instrumental variables (variables that strongly predict the endogenous explanatory variables but do not directly affect the dependent variable–yield, *Striga*, SPAD). As a result, coefficient estimates should be interpreted as indicating association rather than causality. Ridge spacing is the distance between two ridges at each of three locations within a field, measured in centimeters. To avoid edge effects, observations were made that were at least two ridges from the plot border and all locations were at least two ridges apart. The enumerators then randomly chose three locations along a diagonal transect and measured from the center of one ridge to the center of the adjacent one. Total ridge weed biomass was measured in quadrats of 0.5 m by 1.0 m size, at one randomly chosen location per plot. Biomass fresh weight measurements were made in the field, and a subsample from a homogenized sample (weed biomass chopped into ~10-cm size pieces, and mixed thoroughly) was used to determine the wet/dry weight ratio. This measurement was done at harvest, and used as a proxy for the endogenous weed pressure in a field and as an indicator of the effectiveness of weed management.

The variables for intercropping and manure/compost use were all measured as binary variables, and we did not consider the density or the genre of the crop that farmers intercropped with maize. Manure and compost were combined into a single binary variable having a value of one if the farmer applied either one during a given year, and value of zero otherwise. We calculated the amount of nitrogen applied with any fertilizer amendment, based on the farmer reported-fertilizer type (converted as follows, urea is 46:0:0 and the common form of fertilizer locally called NPK is 23:21:0) and amount of fertilizer reported as applied that season. Trained enumerators measured the plot that was fertilized using a built in GPS based area measuring function. In the maize yield model, *Striga* and SPAD were also included as explanatory variables.

In addition to the three models described above, we conducted two sets of analyses to consider if socio-economic indicators are of importance in predicting maize yield: educational level of the household head and total dependency ratio of each household. The analysis was based on models where these two variables were added to the data matrix *X*. With the available data, it was not possible to reject or assert their importance at any reasonable level of significance (e.g 80% credibility or higher). These inconclusive results are included as supporting information (S1 and S2 Figs). These supplemental data show that the regression coefficients of the other variables in *X* were insensitive to whether or not one includes the additional two socio-economic indicators. This is evidence that our regression model was robust. We carried out additional analyses by removing certain variables from *X*, such as ones which may not be significant, and the remaining analyses were largely unaffected; these results are not reported here. They were, however, similar to S1 Fig, which indicates model robustness.

The available data were also used to investigate non-specific household effects, which may be interpreted as pointing indirectly to socio-economic effects, or directly to effects of farmer skill. This analysis used simplified versions of the three linear models, with no predictive factors *X*, pooled all EPA sites together to increase power, and allowed the *y*-intercept parameter *α* to depend on the household identifier. Despite the limited number of datapoints per household (up to 4), we were able to identify nearly 10% of households where such an effect can be detected. The results of this household analysis are provided in the Results section.

Each of the three aforementioned models was specified in the simple linear framework, with appropriate logistic modifications in the case of the the *Striga* model, to distinguish between incidence of *Striga* and levels of *Striga*. Such a linear framework can be considered as a first-order approximation for each response, with the understanding that it would not be possible to distinguish, in a statistically significant way, between each of these models and other models with non-linear features. This paper did not delve into the consideration of higher complexity models, since they lied beyond the scope of our dataset and thus of our analysis. As such, the three structural models were uniquely characterized by their respective response variables and explanatory variables. Because the models had the same set of explanatory variables, an identification problem may exist, whereby we were unable to uniquely identify the parameters of the structural model. While the problem could in theory be remedied by having at least one explanatory variable be different in each structural model, in practice this is not possible given the limited amount of data in this study.

For each model, the set of explanatory variables was chosen from an agromomist perspective, to be consistent with domain experts’ beliefs about what factors may influence yield, SPAD, and *Striga*. Slightly streamlined yield models were also investigated, where either SPAD or *Striga* were removed. It turned out that these reduced models resulted in decreasing explanatory power for all variables. These results were not reported, since their empirical statistical power, which is easily assessed via the Bayesian posterior distributions on regression coefficients, is lower.

In sum, this study focused on biophysical determinants, yet there is more to be explored in the future regarding socio-economic determinants.

#### Bayesian analysis

The Bayesian approach allows background knowledge from domain specialists to be incorporated into the analysis, as a type of participatory model building, improving the accuracy and credibility of the estimations [19]. Second, Bayesian statistics show full probability distributions (posteriors) for every model parameter, providing more information than point estimates like means and variances in classical frequentist statistics. P-values can be computed in a Bayesian analysis, with more power and flexibility in assessing the significance of explanatory variables [27]. This is also a way to avoid common misinterpretations of p-values, a documented problem in the application of frequentist statistics [28, 29].

A third advantage of Bayesian analysis is in increased statistical power for data-limited studies. Many papers have shown the benefits of Bayesian statistics in the context of smaller datasets [30–33]. There is an often quoted but rarely if ever formally cited rule of thumb in Bayesian statistics, by which the number of parameters (or degrees of freedom) that one can estimate reliably (e.g. with credibility level higher than 90%) in a linear model, is a third of the total number of datapoints, compared to a tenth in ordinary frequentist linear regression. See [32] Section IV.B for a description of this phenomenon.

To evaluate determinants of maize yield, we compared the effects of each explanatory variable on the response variable in any one of our three models (yield, *Striga*, SPAD). Because all variables in the models have been standardized, the estimated regression coefficients of each variable gave a magnitude of influence (beta weights) to find out which among the explanators of *Y* have the strongest effect. All the figures in this article present the values and credible intervals of the regression coefficients via this simple magnitude metric. These magnitudes can also be compared with the posterior mean of the noise term (*σ*). Usually, *σ* is quite large relative to a single regression coefficient, but one should add the absolute magnitudes of several of the explanatory variables for a more meaningful comparison, since the statistical error needs to be compared to the strength of all the explanatory factors combined.

We implemented Bayesian analysis using the package PyMC3 built into the object-oriented programming language Python. This package provides a procedure to estimate the posterior distribution of our model parameters (regression coefficients and error terms) by implementing a sampling procedure for these distributions. It uses the commonly used Gibbs sampler to produce these samples, with a burn-in period of 500 initial samples, and an additional 10,000 iterations with two independent Monte Carlo Markov Chains (MCMC) after each burn-in. The posterior distributions from these two chains are then compared to gauge the procedure’s convergence. Since we did not have prior knowledge on the distribution of the parameters, in this simple linear setting, we used the classical prior distributions, which are weakly-informative [34]: the standard normal distributions for the regression coefficients and inverse-gamma distribution for the error term. These prior choices present numerical advantages in terms of conjugacy [27, 34]. The reason for choosing standard (mean-zero, unit variance) normal distribution as opposed to other means and variances, is because we work with standardized variables as explained previously. We monitored the convergence of the procedure by keeping track of the discrepancy between the two aforementioned chains, using Python’s R-hat statistic a widely accepted convergence diagnostic statistic. All the R-hat values were below the threshold of 1.1 which is considered acceptable, implying that the chains successfully converged, producing excellent approximations of our parameters' estimates and their credible intervals.

## Results

### Descriptive statistics

Table 1 provides descriptive statistics for the model variables by study location. Mean values for rainfall were consistent with earlier characterization of Golomoti as a low rainfall, marginal site [14]. The other locations differ in terms of mean rainfall for March, with the high potential site (Linthipe) having the highest rainfall level.

**Table 1:** Descriptive statistics of the Bayesian model variables, mean and standard error, are presented by location. Household and plot level management practices and biophysical observational data were collected through a mid-season and a harvest survey, conducted in 2015 and in 2016.

Variable	Golomoti	Linthipe	Kandeu	Nsipe
	Mean (SE)			
**Observations (#)**	312	282	298	305
**Environment**				
**Rainfall, December to February (mm)**	563.0 (78.0)	624.5 (27.7)	629.5 (94.6)	636.8 (132.8)
**Rainfall, March (mm)**	78.6 (26.8)	128.9 (42.0)	102.8 (26.5)	116.8 (25.4)
**Rainfall,** **April and May (mm)**	25.2 (10.4)	43.8 (11.6)	39.1 (9.1)	44.4 (8.8)
**POXC (mg C/kg soil)**	278.9 (152.43)	466.9 (220.5)	390.41 (191.15)	340.70 (160.22)
**Soil ph**	6.56 (0.61)	6.09 (0.46)	6.10 (0.53)	6.32 (0.61)
**Crop performance**				
**Maize yield (kg/ha)**	1567.44 (1039.3)	2636.3 (1526.5)	2069.4 (1471.5)	2320.9 (1452.9)
**SPAD**	41.20 (8.85)	46.98 (7.15)	46.00 (8.62)	41.84 (8.11)
**Management practice**				
**Maize spacing (m)**	0.897 (0.11)	0.927 (0.11)	0.970 (0.13)	0.914 (0.14)
**Weed biomass (kg/m**^**2**^**)**	0.183 (0.16)	0.159 (0.15)	0.201(0.15)	0.246 (0.16)
**Fertilizer use (0/1)**	0.67 (0.47)	0.80 (0.38)	0.85 (0.36)	0.81 (0.38)
**Striga (0/1)**	0.22 (0.42)	0.30 (0.46)	0.16 (0.37)	0.28 (0.45)
**Intercrop (0/1)**	0.66 (0.47)	0.77 (0.42)	0.74 (0.44)	0.60 (0.49)
**Compost (0/1)**	0.41 (0.49)	0.44 (0.50)	0.31 (0.46)	0.27 (0.45)

Soils were generally marginal at Golomoti sites, as evidenced by low mean values for soil active carbon (POXC), consistent with earlier reports [14]. Soil active carbon was highest in Linthipe. Soil pH varies little by location, and mean values are consistent with moderate acidity, and thus non-limiting pH conditions for the crops grown.

Crop response data includes maize yield and leaf nitrogen content, as indicated by SPAD values. Average maize yield is lowest in Golomoti, followed by Nsipe and Kandeu, and highest in Linthipe. Maize SPAD values followed a similar but not identical pattern: low in Golomoti and Nsipe, and high in Kandeu and Linthipe.

*Striga* incidence and weed biomass was distributed across EPAs with no clear spatial pattern. Farmers reported *Striga* problems on about 16–30% of sampled fields, and from 0.18 to 0.25 kg/m2 dry weight weed biomass remaining in the field at harvest (Table 1). The latter is an indicator of how effective farmer weed management is, although the endogenous infestation levels of weeds at a site could also contribute to observed presence.

Overall, fertilizer use was lower on the Golomoti sites than the other EPAs. This is consistent with the intuition that farmers in marginal environments have less motivation to invest in their land and crops. Fertilizer use was similar in Kandeu, Linthipe, and Nsipe. Compost application was higher in Linthipe and Golomoti than in Kandeu and Nsipe. As expected, intercropping was more frequently practiced in Linthipe and in Kandeu, locations where many farmers grow bean and cowpea in mixed stands with maize [14]. Average plant spacing was lowest in Golomoti and Nsipe (~0.9 m between ridges), and highest in Kandeu (~1 m between ridges).

### Bayesian model results

Statistical significance was determined in a linear Bayesian context. An explanatory variable (such as SPAD) in a linear model for a response variable (such as yield) is statistically significant at 95% Bayesian credibility if its regression coefficient has a posterior probability of being on one side of zero which exceeds 95%. This is true as soon as the 95%-credibility intervals (CI) of that variable’s regression coefficient lies on one side of zero. Strictly speaking, any time a variable’s 95% CI fails to lie on one side of zero, one may then accuse it of not being statistically significant. This is a steep threshold to apply in most cases, since a variable with, for example, 90% Bayesian credibility, still holds some predictive information. In this paper’s analysis, however, when an explanatory variable fails to be significant in its association to the model’s response variable, this failure occurs at a much lower level than 90%, as seen in the forest plots (S2 Fig). The size of an association needs to be distinguished from its significance. For variables which are significant, their size, or intensity, of this significance is measured by the variable’s regression coefficient’s posterior mean.

#### Maize yield

**Fig 1:**
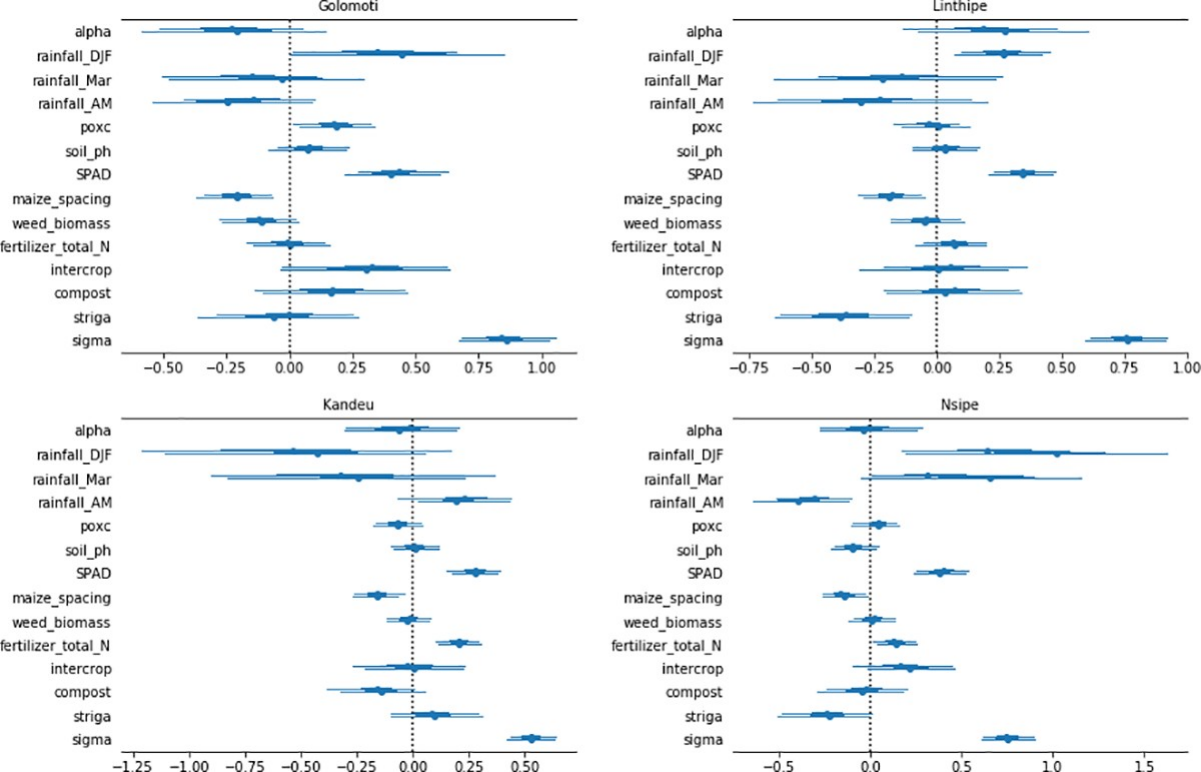
Bayesian regression model results for 95% credible intervals associated with drivers of maize yield for four EPA locations in Central Malawi.

Fig 1 presents the 95% credibility intervals of the determinants of maize yields at the different sites. Across sites, maize yield was positively associated with SPAD and negatively associated with plant spacing; the magnitude of the coefficients for SPAD were particularly large. Fertilizer quantity was positively associated with SPAD, which in turn was highly predictive of maize yield. There was also a small but positive direct association between fertilizer and maize yield, for Kandeu and Nsipe. Yield was not consistently influenced by soil pH, weed biomass, intercropping, or March rainfall.

The *α* values in Fig 1 differ markedly from each other by EPA, which suggests that location has an important influence on maize yield, independently of other factors in the model. Since those factors were capable of exacerbating differences in maize yield by location, we turn now to consider locational differences.

First, rainfall levels are only statistically significant factors for yield in Linthipe and Nsipe. In both EPAs, early season rainfall (i.e., December to February) positively correlated with maize yield. In Nsipe, high rainfall in April/May was associated with low maize yield, perhaps a reflection of late-season disease harming the crop, such as Fusarium ear rot [35].

Secondly, soil active carbon and compost application were positively associated with maize yield at the marginal site, Golomoti. Third, in Linthipe and Nsipe, striga had a large negative effect on maize yield, whereas it was not significantly associated with maize yield in Golomoti and Kandeu. Striga incidence was similar across locations (Table 1).

Overall, the results of the Bayesian maize-yield model indicate that maize leaf nitrogen (SPAD) and striga were the strongest determinants of maize yield at the study sites, while early and late rainfall are significant predictors of yield at some sites. As striga and SPAD can be directly influenced by farmer behavior, we estimated separate Bayesian models to uncover the drivers of these two critical inputs to maize yield.

### SPAD

**Fig 2:**
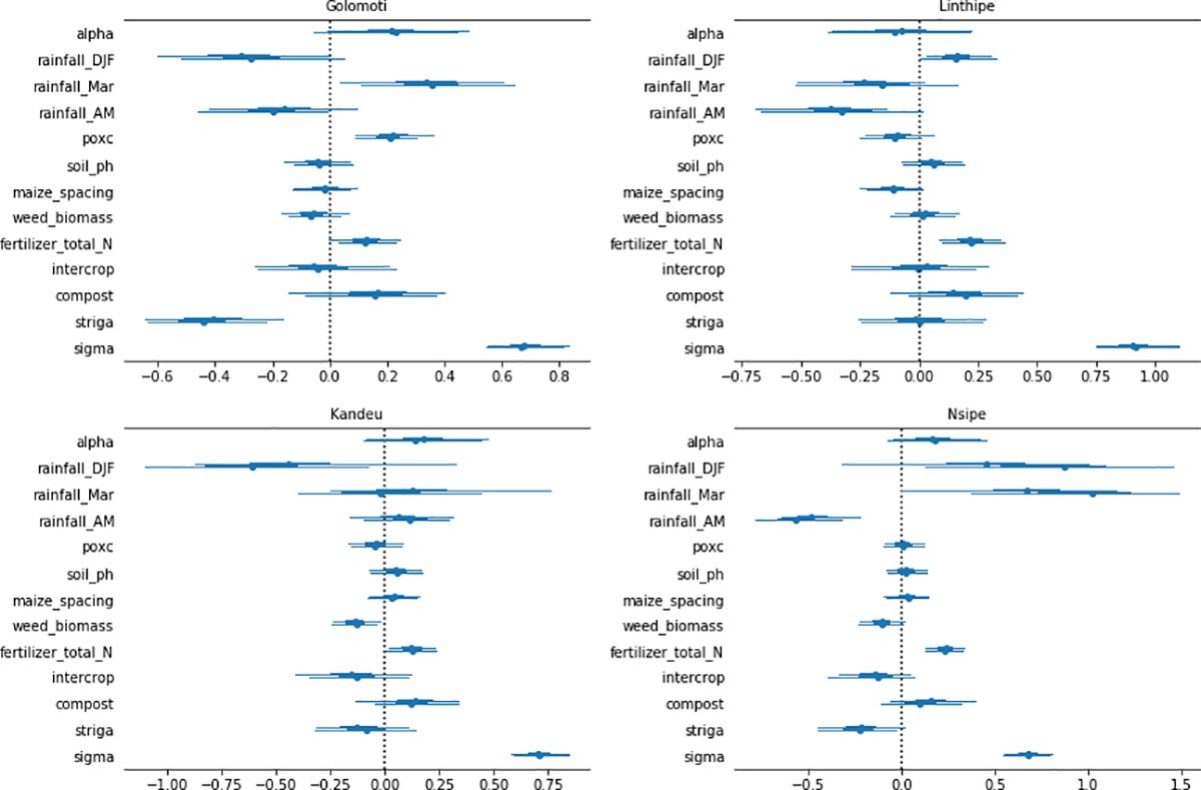
Bayesian regression model results for 95% credible intervals associated with drivers of SPAD for four EPA locations in Central Malawi.

The Bayesian regression model identified three main determinants of SPAD: rainfall, poxc, and application of fertilizer or manure (Fig 2). Rainfall was generally found to be influential on SPAD, except in Kandeu, where rainfall variables were not statistically significant. In Golomoti, SPAD increased with March rainfall, whereas in Linthipe and Nsipe it was December-February rainfall that had a positive influence on SPAD. Also in Nsipe, a negative association with SPAD was observed for late season rainfall.

Fertilizer was an important predictor of SPAD at all sites except the dry, marginal site of Golomoti (Fig 2). In marginal sites, plant growth and response to fertilizer was often limited by insufficient soil moisture, thus fertilizer application doesn’t necessarily lead to plant uptake of nitrogen or yield response. Indeed, our maize yield model results also showed a lack of response to fertilizer in Golomoti (Fig 1). Manure application and the soil property poxc were positively related to SPAD at this site. Fertilizer effects on both SPAD and yield were highly variable, however, suggesting the need to improve the effectiveness of fertilizer applied. The magnitude of the fertilizer effects on SPAD were higher in Kandeu and Nsipe than Linthipe.

### Striga

**Fig 3:**
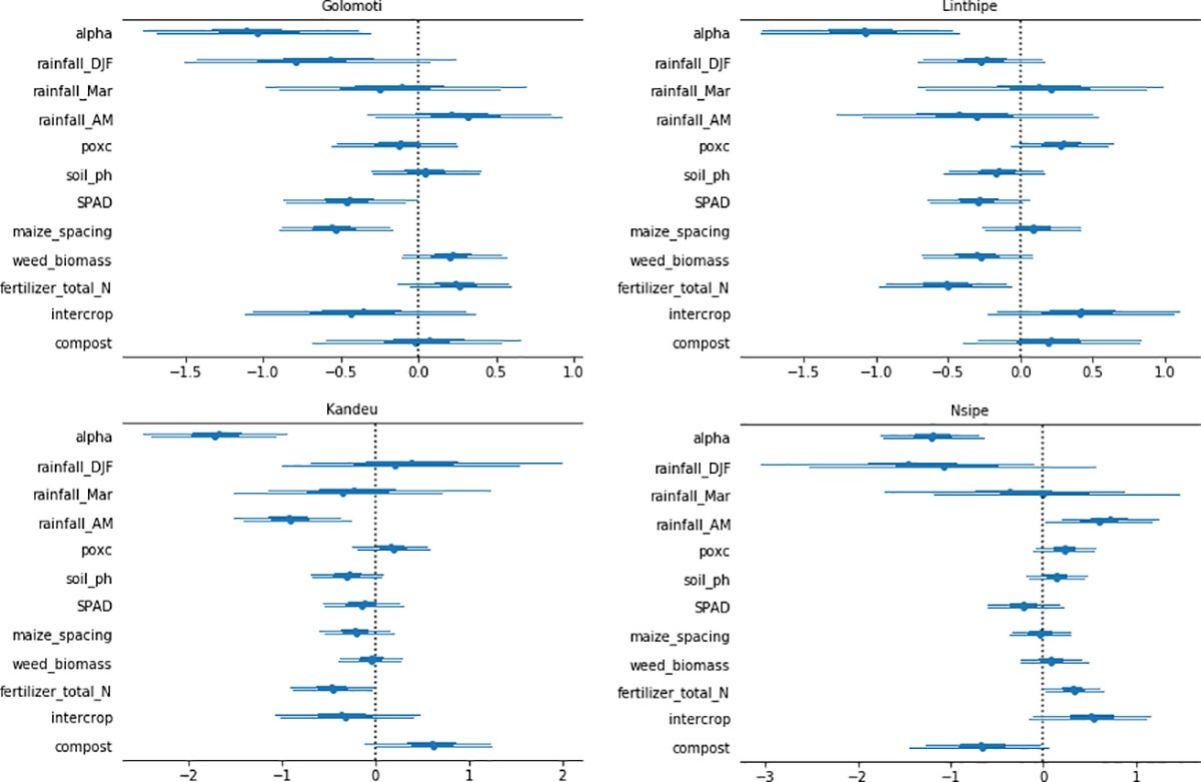
Logistic regression results for 95% credible intervals associated with drivers of striga prevention for four EPA locations in Central Malawi.

**Fig 4:**
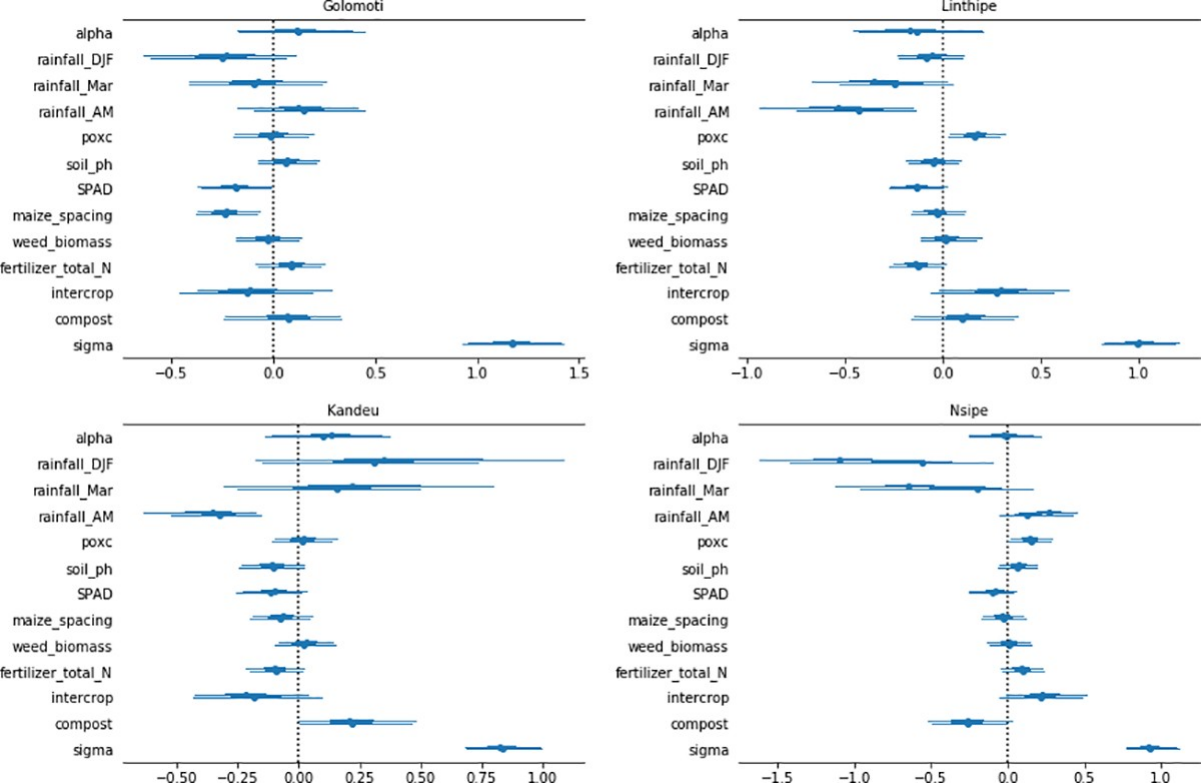
Bayesian regression model results for 95% credible intervals for the factors associated with striga control for four EPA locations in Central Malawi.

Factors that influenced striga incidence (Fig 3) and level of striga infestation (Fig 4) are presented here, as striga was found to be an important negative driver of yield. The model can be thought of as a two-step procedure. First, the zero and non-zero values provide two alternatives which allow one to estimate the influence of the presence or absence of striga via logistic regression, which speaks to the possibility of prevention. Next, when conditional on the presence of striga, reverting to an 8-point scale of quantitative values of striga, ordinary linear regression is used, which is linked to the effectiveness of striga control. Having a large number of observations equal to zero (known as zero inflation) may induce biased results [36]. The proposed two-step analysis mitigates this problem, since the standard linear regression model is relative to the data with non-zero striga values. The striga infestation level model assessed field observations of striga at integer values from 0 to 8, with 0 indicating the absence of striga and the values from 1 to 8 revealing the level of striga infestation (based on mid-season plot observations). As stated, the logistic regression model can reveal possible striga prevention measures, whereas the 1-8-levels model provides insights on possible striga control measures.

Among the farmer management factors and soil properties, most were either not significant or of small magnitude in relationship to striga. There was some evidence of soil fertility amendments being useful in prevention, as fertilizer use was assocated with striga absence (as reported by farmers) in Kandeu and Linthipe. Fertilizer was also an apparent control factor in Linthipe, as it was a predictor of low striga incidence at this site. Compost was associated with both striga absence and low incidence in Nsipe, with contrasting results observed in Kandeu. At the marginal Golomoti site, fertility amendments had no striga control benefits, and the only farmer management effect was wide ridge spacing, which was negatively correlated to striga incidence and intensity. Overall the results show intercropping is generally neither helpful nor harmful to striga prevention and control. The one exception was Linthipe where intercrops were associated with farmer-reported striga problems.

### Farmer (household) effect

The farmer-effect study described earlier used each of the three models with indicator (dummy) household variables to help determine whether households are predictive of yield, SPAD, or striga. With 306 households, and up to four data points per household (two maize plots surveyed per household in 2014 and 2015), we found that in most cases, it was not possible to determine whether there is a connection between any particular household and their corresponding four datapoints. However, in roughly 10% of cases, some statistical significance was extracted, meaning that the variability among the four datapoints of each such household is almost certainly not due to chance alone. Moreover, the effect was most likely to identify a favorable household environment. Specifically, setting the significance level at 5%, we computed the number of households for which the corresponding regression intercept *α* is away from zero with Bayesian posterior probability at least 95%. The results are summarized in Table 2. A sample of details of the data analysis output for the yield model, including 95%-credibility interval for each household, and corresponding forest plots, are given in the supporting information section, S2 Fig.

**Table 2:** Number and proportion of households for which the *y*-intercept regression coefficient *α* is significantly non-zero at the 95% credibility level.

Model	Number (%) of households with positive effect	Number (%) of households with negative effect
**Yield**	23 (**7.5%**)	5 (**1.6%**)
**SPAD**	11 (**3.6%**)	7 (**2.3%**)
**Striga**	26 (**8.5%**)	0 (0.0**%**)

For the yield model, the total number of significant households exceeded 9%. Interestingly, among these households, there were far more cases where the yield was higher than expected due to chance alone, than cases where it was lower (7.5% against 1.6%). In other words, when the data points towards a farmer having a significant effect on yield, the odds are about 5:1 that this was a skilled farmer with high maize yields.

For the two other models, similar results hold, at the 5% significance level, to a slightly lesser extent. For the SPAD model, about 6% of households had SPAD levels which cannot be explained by chance, and among those, the odds of having good SPAD against low SPAD was about 3 to 2. For the Striga model, there were no cases where one could say that a farmer was likely to be avoiding striga entirely, while 8.5% of farmers were likely to be associated with a Striga problem.

The results on striga may seem surprising: we cannot determine any farmer with the skills or the knowledge of practices to be superior to others in preventing striga. This conclusion is not to be taken as a discouraging fact. Rather, it reflects that over two thirds of all plots in the study area, i.e. a large proportion, were striga-free. Thus, while one third of plots being infected with striga reaches epidemic levels, from the standpoint of statistical power, having two thirds of plots without striga makes its absence so prevalent that the 306 households cannot identify anyone with unusual striga-prevention skills. Readers are referred to the full striga model for more precise and statistically significant recommendations on striga.

## Discussion

The plot-level longitudinal dataset and Bayesian statistical models reported here place biophysical constraints in sharp focus in the analysis of what influences maize yield in Malawi. Some of the identified drivers of maize yield, notably SPAD, striga, and sub-seasonal rainfall patterns, have strong effects with very high credibility. Overall, nitrogen nutrition was a key determinant of yield, consistent with much of the literature on small scale, mixed maize production systems [1, 6]. Yet, fertilizer application did not necessarily result in improved leaf nitrogen (SPAD values) or in subsequent maize yields. Thus the results highlight the challenges to ensuring effective nitrogen uptake and translation into grain, particularly in marginal environments. In concurrence, a study in Western Kenya found that maize yield response to nitrogen fertilizer application is low when soil organic matter is low [37].

Fertilizer in Central Malawi was generally associated with high plant nitrogen tissue (SPAD values) at all but the marginal location of Golomoti. This is consistent with recommending to farmers the use of manure or legume rotations at marginal sites, to build soil active organic matter and thus soil nitrogen supply capacity [38]. Integrated management of soil fertility combining manure and fertilizer has been shown previously to be highly effective and profitable for raising maize yields [39]. Soil organic carbon fractions tend to be sensitive indicators of crop response at lower values, as we observed here, as Golomoti had 20 to 40% lower levels of active soil carbon compared to the other sites.

Rainfall was an important factor influencing maize yield, particularly early-mid season rainfall (Fig 1). This supports a previous study in Malawi that found a positive association between seasonal rainfall and maize yield based on an econometric analysis of farm-level data [39]. At two sites we found a negative relationship of late season rainfall to yield, and this negative effect was also observed for SPAD. This could reflect a leaching problem, with rainfall inducing soil inorganic nitrogen losses and thus limiting nitrogen availablity during the critical maize grain filling period—which requires high nitrogen availability [40]. It however could also be related to Fusarium or other infections of the corn ear, induced by a late season moist environment causing grain spoilage and thus yield loss [31]. Ours is the first study that we know of that used household and focal plot survey data to produce this type of differentiated conclusions, based on sub-seasonal data.

The findings highlighted the large negative effect of the parasitic weed striga on maize yield. Although weed biomass alone had no discernable effect, striga was a strong negative determinant of maize yields in Linthipe and Nsipe. These are relatively mesic locations with overall medium to high maize yields, compared to Golomoti and Kandeu. It is suprising that the negative effects of striga are only observed at farms in Linthipe and Nsipe, as the presence of this parasitic weed is common throughout the study sites and is indeed ubiquitous in Central Malawi [41]. Our finding of a highly negative impact of striga, in areas where yield potential is otherwise high, indicates a barrier to agricultural production that has been largely overlooked by agricultural research and policy makers, where the focus has often been on subsidized access to hybrid maize seeds and fertilizers. A need revealed by this study is that of effective and affordable means of striga prevention and control.

In Malawi, farmers rely primarily on hand weeding for striga control, which appears to be ineffective largely due to the parasitic nature of this weed—thus maize growth suppression has already occurred by the time hand weeding is done [42]. We explored the utility of fertilizer and manure/compost application for striga prevention and control, based on an extensive previous literature linking low nitrogen soils to higher striga incidence [43–45]. Similarly, farmers in a recent Malawi survey ranked manure application as the best option for striga control [46]. Given the observed differences in soil fertility and fertilizer use across the study EPAs, we expected higher striga infestation in Golomoti vs. the other areas, yet there is no evidence of this. Instead what we observe is widespread presence of striga and lack of uniformity in what works for prevention and control. For example, chemical fertilizer is found to be an important factor for striga prevention and control, but only in Kandeu and Linthipe. Manure/compost appears to have merits for reducing striga problems in Nsipe only. In short, study findings suggest a complex mode of action with difficult-to-predict reactions of maize to striga presence and striga to control measures. Another study from Malawi similarly found complex relationships between soil fertility and striga: two years of on-farm observations and participatory research revealed that early application of fertilizer helped maize plants overcome early effects of striga attachment, whereas late application of fertilizer was associated with worse striga [47].

Overall, the results from this study call for area-specific recommendations. The Malawi government recommendations for hybrid maize production have focused primarily on N fertilizer rate, which is 69 kg N ha^-1^ throughout most of the country [48, 49]. We found evidence that shows targeting complementary investments and timing of application would add value. For example, maize production at the mesic site would benefit from early, judicious use of fertilizer for striga control, and for nitrogen nutrition benefits. Whereas for the marginal site, response was markedly different, with no fertilizer or striga drivers observed on yield, and instead soil active carbon and compost are positive determinants of yield, and thus recommended practices should focus on soil organic matter management.

Soil carbon accumulation provided important environmental services at all sites; however, at marginal sites the benefits from incremental gains in soil carbon were high, from stable production over time and space, to substantial gains in nitrogen efficiency, and at one site, suppression of striga. Compost preparation, and utilization at modest amounts, had beneficial effects at some sites, with gains in plant health as indicated by nitrogen status and striga suppression. No effects of compost were observed at most sites during the modest (three year) timeline of the project, yet when a site is marginal, and during a poor rainfall season, this management practice is one of the few consistently beneficial practices. With little to no downside, this study highlights the role that government policies, extension and educational efforts by civil society can all have in building appreciation for compost benefits, particularly in dry environments.

## Supporting information

S1 Fig Bayesian regression model results for 95% credible intervals associated with socioeconomic and agro-ecological drivers of maize yield for four EPA locations in Central Malawi. (TIF)

S2 Fig Forest plots for 95% credibility intervals for the y-intercepts for 306 households in the yield model. (TIF)
