# Supplementary figures and images for: A Bayesian analysis of longitudinal farm surveys in Central Malawi reveals yield determinants and site-specific management strategies
Source: PLoS One. 2019 Aug 8;14(8):e0219296. doi: 10.1371/journal.pone.0219296 (PMC6687183; doi:10.1371/journal.pone.0219296)

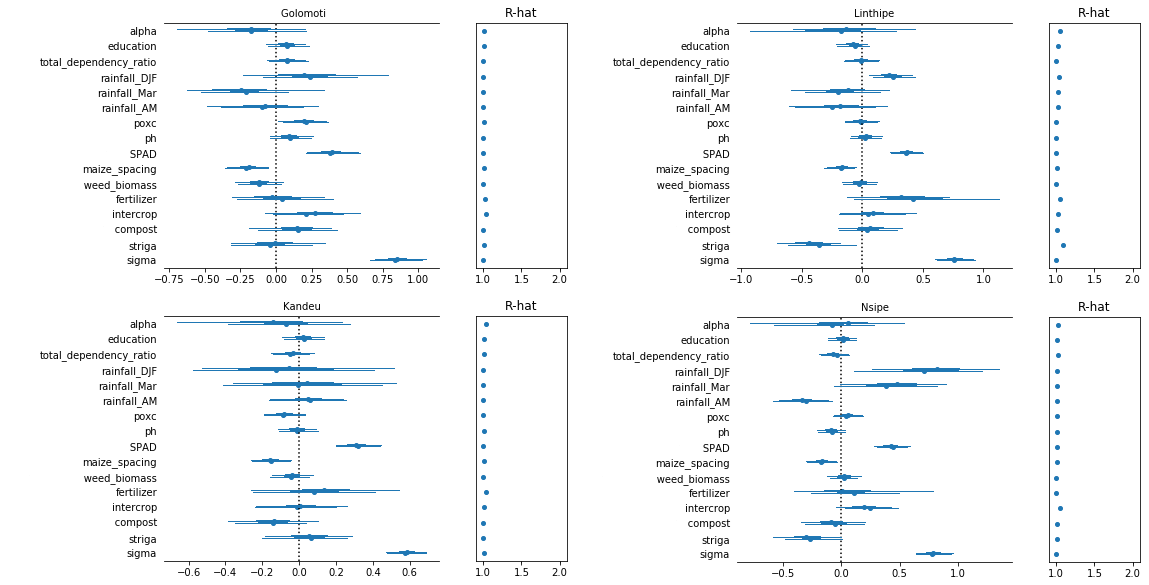

Supplement: S1 Fig — (TIF) [file pone.0219296.s001.tif]

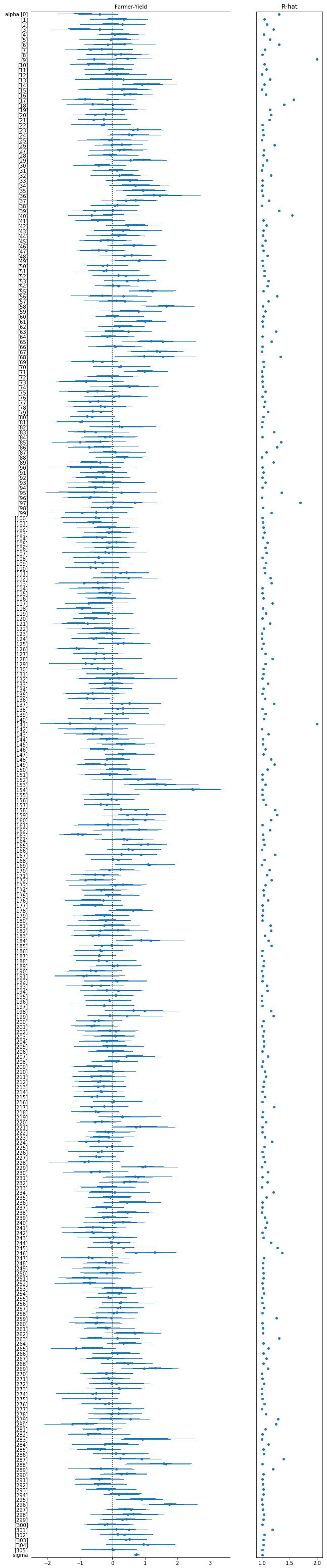

Supplement: S2 Fig — (TIF) [file pone.0219296.s002.tif]
